# Association between the Mediterranean Diet Index and self-reported Gingival Health Status Indicators in a population of Chilean adults: a cross-sectional study

**DOI:** 10.1590/1678-7757-2023-0100

**Published:** 2023-07-03

**Authors:** Gustavo SÁENZ-RAVELLO, Loreto MATAMALA, Patricia CISTERNAS, Jorge GAMONAL, Patricia HERNÁNDEZ, Nidia Castro dos SANTOS, Ke DENG, Mauricio BAEZA

**Affiliations:** 1 Universidad de Chile Facultad de Odontología Centro de Epidemiologia y Vigilancia de las Enfermedades Orales Santiago Chile Universidad de Chile, Facultad de Odontología, Centro de Epidemiologia y Vigilancia de las Enfermedades Orales (CEVEO), Santiago, Chile.; 2 Universidad de Chile Facultad de Odontología Departmento de Odontología Conservadora Santiago Chile Universidad de Chile, Facultad de Odontología, Departmento de Odontología Conservadora, Santiago, Chile.; 3 Universidad de Chile Facultad de Odontología Santiago Chile Universidad de Chile, Facultad de Odontología, Santiago, Chile.; 4 Universidade Guarulhos Divisão de Pesquisa Odontológica Guarulhos SP Brasil Universidade Guarulhos, Divisão de Pesquisa Odontológica, Guarulhos, SP, Brasil.; 5 The Forsyth Institute Cambridge MA United States The Forsyth Institute, Cambridge, MA, United States.; 6 The University of Hong Kong Faculty of Dentistry Division of Periodontology and Implant Dentistry Hong Kong SAR China The University of Hong Kong, Faculty of Dentistry, Division of Periodontology and Implant Dentistry, Hong Kong SAR, China.

**Keywords:** Mediterranean diet, Gingival diseases, Self-report, Internet-Based Intervention

## Abstract

Despite the recognized impact of diet on non-communicable diseases, the association between the Mediterranean diet and periodontal diseases is still uncertain. This study aimed to determine the association between adherence to the Mediterranean Diet Index (MDI) and self-reported gingival health status in Chilean adults, exploring the feasibility of using validated web-based survey questionnaires.

**Methodology:**

Cross-sectional data were collected from a representative sample of a population of Chilean adults (18-60 years old) using a low-cost and time-saving methodology. By the PsyToolkit platform, anonymous survey data were downloaded and analyzed in bivariate (crude) and backward stepwise selection multivariate logistic regression models adjusted for sociodemographic determinants, smoking, and dental attendance using STATA 17. Odds ratios (OR) [95% confidence intervals] were estimated.

**Results:**

In total, 351 complete statistical data were mostly obtained from female university students who had never smoked and reported having visited a dentist in the previous year. Multivariate regression models showed an association between MDI and very good/good gingival health status (OR 1.18 [95% CI 1.04-1.34], p=0.013), absence of bleeding on toothbrushing (OR 1.12 [95% CI 1.01-1.25], p=0.035), and absence of clinical signs of gingival inflammation (OR 1.24 [95% CI 1.10-1.40], p<0.001), after controlling for age, sex, educational level, smoking, and dental attendance.

**Conclusions:**

We associated adherence to the Mediterranean diet with better self-reported gingival health status in a population of Chilean adults in an entirely web-based research environment. Longitudinal studies with random sampling are required to establish the effect of diet on gingival and periodontal health. Nevertheless, this evidence could contribute to the design of low-cost surveillance programs to reduce the burden of periodontal disease and related “common risk factors”.

## Introduction

Non-communicable diseases (NCDs) are highly prevalent chronic diseases that strongly contribute to the global burden of disease and are one of the leading causes of mortality worldwide.^1^ Periodontal diseases (PDs) are NCDs due to sharing pathogenic mechanisms that link them from a preventive and therapeutic perspective, and sociodemographic determinants including age, sex, and educational level, as well as risk factors such as smoking, sedentary lifestyle, and obesity.^2^ Plaque-induced gingivitis is an inflammatory response of gingival tissues resulting from bacterial plaque accumulation located at and below the gingival margin. If supra- and subgingival plaque is not removed, an inflammatory immune response is triggered in a susceptible host, which can lead to progressive destruction of tooth-supporting tissues, causing periodontitis and subsequent tooth loss.^3^Recently, studies have investigated the potential effects of nutritional supplements on the development and progression of PDs.^4^ Although the preventive role of diet in the course of NCDs has been widely documented,^1^ knowledge regarding its role in PDs is lacking.

Diets rich in vegetables have high anti-inflammatory characteristics due to their high content of vitamins, flavonoids, polyphenols, nitrates of vegetable origin, omega-3, and essential minerals such as calcium, magnesium, and zinc, preventing cardiovascular diseases, the incidence of cancer, neurodegenerative diseases, and type 2 diabetes mellitus, significantly reducing C-reactive protein, a marker associated with complications and mortality from NCDs.^5^ These nutrients have shown significant improvements in the clinical and microbiological parameters of periodontal disease, thus suggesting a promising preventive and therapeutic value.^4^ However, dietary patterns are a broader picture of food and nutrient consumption and may be more predictive of disease risk than individual food or nutrients.^6^

The Mediterranean diet is primarily a plant-based dietary pattern that includes the daily intake of whole grains, olive oil, fruits and vegetables, legumes, nuts, and a small quantity of animal protein, with fish and seafood as the preferred animal protein.^7^ Although the available evidence shows a potentially beneficial effect of the Mediterranean diet on gingival health status, it is still unable to allow a high certainty.^4^ The risk of bias is high due to measurement bias given the use of non-standardized dietary indices.^4^ Furthermore, the effects of sociodemographic covariates that could influence adherence to the Mediterranean diet and healthy oral behavior have not been considered.^4^ Therefore, further studies in a real context are required to clarify the association between the Mediterranean diet and gingival health indicators.

One way of inexpensive epidemiological study for PDs and nutrition surveillance is self-reporting measures and indices, especially useful when clinical examination is unfeasible, that is, in a low-resource setting or when clinical measurements are unfeasible, such as during the COVID-19 quarantine. These measures have demonstrated acceptable sensitivity and reliability in the evaluation of PDs and dietary patterns.^8 - 10^ Questions about gum health/disease, gum treatment, loose teeth, bone loss, tooth appearance, floss, and mouthwash use showed moderate to high accuracy in identifying gingivitis and periodontitis, with an area under the receiver operating characteristic curve (ROC) of 0.837.^10^ Moreover, self-reported measures combined with age and tobacco smoking showed excellent performance for identifying Stages III/IV periodontitis with a high ROC of 0.953, a sensitivity of 95.7%, and a specificity of 89.0%.^10^ Furthermore, bleeding on toothbrushing may be a promising sentinel sign of gingival inflammation, which showed low to moderate accuracy in discriminating periodontitis and gingivitis from periodontal health, with a sensitivity of 37.1% and 61.3% and a specificity of 84.8% and 84.4%, respectively.^9^ In Chile, self-reporting of bleeding on toothbrushing has 38.9% sensitivity and 85.9% specificity, with a ROC of 0.71, to classify individuals with gingivitis.^11^

This cross-sectional study aimed to determine the association between adherence to a Mediterranean diet and self-reported gingival health status in an adult population living in Chile, exploring the feasibility of using validated web-based survey questionnaires. Our hypothesis was that there is an association between the study variables, after adjusting for age, sex, educational level, dental visits in the last year, and smoking, when assessed entirely in a web-based research environment.

## Methodology

This cross-sectional, analytical, observational study was approved by the institutional review board of the Institute for Research in Dental Sciences of the University of Chile (DIFO CODE:2020/15) and was conducted in accordance with the Helsinki Declaration of 1975, as revised in 2013. This study complies with the STROBE^12^ and CHERRIES^13^guidelines.

To reduce the bias, several methodological recommendations^14^ were considered in the development of the research protocol, such as using locally validated questionnaires hosted in platforms compatible with several devices (e.g., smartphone, computer, and tablet), restricting survey length to below 13 minutes and provide anonymity, to reduce the “Hawthorne effect”. Furthermore, to reduce recall bias, the recommendation of Bartha, et al.^15^(2022) was followed, including the locally validated version of the Mediterranean diet score, since the use by patients is easy, instantly interpreted by users, and demonstrated comparable values for nutrient intake as assessed by the 24-hour dietary recall and Food Frequency Questionnaire: the Mediterranean diet index.^16^ Moreover, the researchers tested the usability and technical functionality of the web questionnaire before disseminating the survey, reporting no problems or difficulties in its development.

### Setting and Participants

Data were obtained by a voluntary self-reported anonymous survey available on the PsyToolkit platform^17 , 18^ for adults aged between 18 and 60 years from the community of the University of Chile. The inclusion criteria were being at least 18 years old and having the time and willingness to answer the survey. Exclusion criteria were based on well-known biological confounding factors for gingival inflammation such as pregnancy and use of anti-inflammatories or systemic antibiotics.^19^ Other potential confounders were included, analyzed, and/or properly adjusted in the final model to achieve greater external validity.

**Figure 1:**
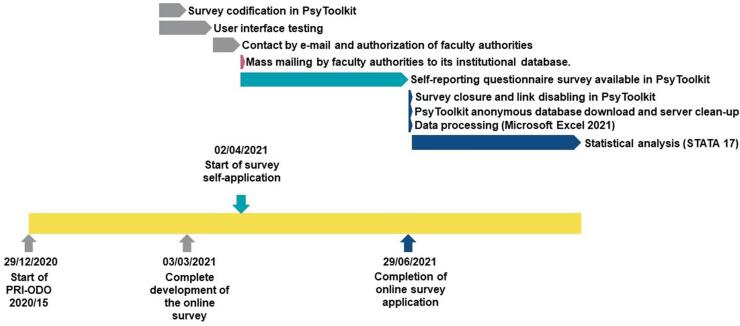
Flowchart illustrating the study process

Dissemination was channeled by a formal letter to the corresponding authorities of the Faculty of Medicine, School of Public Health, Institute of Nutrition and Food Technology, Faculty of Chemical Sciences and Pharmacy, and Department of Theater of the University of Chile, who sent the survey link to their institutional database between April 2 and July 29, 2021. Written informed consent was obtained from all participants at the beginning of the survey, and it described the length of time of the survey (~5 minutes) and the progress throughout the survey (progress bar), which data were stored (anonymous) and where and for how long, who the main researcher was, and the objective of the study. The incentive to answer the survey was to immediately know the results of the respondent (regarding the Mediterranean diet index score). A sample of the user interface is available as supplementary material (Supplementary file 1 and 2). Figure 1 shows the whole study process.

### Variables and sources of information

**Figure 2:**
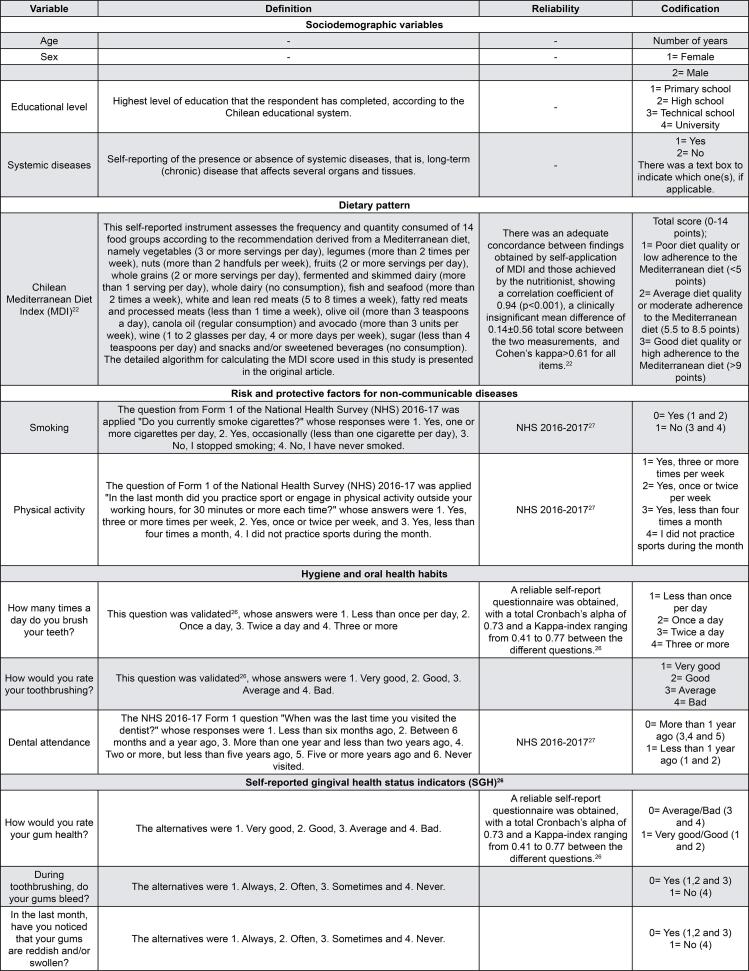
Categorization and operationalization of study variables

Individuals self-reported two validated instruments, the Mediterranean diet index (MDI)^16^ and self-reported gingival health status (SGH),^20^ sociodemographic variables, risk and protective factors, and hygiene and oral health habits, on an anonymous PsyToolkit platform.^17 , 18^ Figure 2 shows details of each measure.

### Sociodemographic variables

Individuals’ age, sex (male or female), and educational level (primary school, high school, technical school, or university) were recorded. The presence or absence of systemic diseases, that is, long-term (chronic) diseases that affect several organs and tissues, were also self-reported.

### Dietary assessment

The Chilean MDI is an 14-item instrument^16^ based on a previous Mediterranean eating score that was adapted to Chilean dietary habits and assesses the frequency and quantity consumed of 14 food groups according to the recommendation derived from a Mediterranean diet, namely vegetables (3 or more servings per day), legumes (more than 2 times per week), nuts (more than 2 handfuls per week), fruits (2 or more servings per day), whole grains (2 or more servings per day), low fat and fermented dairy (more than 1 serving per day), whole fat dairy (no consumption), fish and seafood (more than 2 times a week), white and lean red meats (5-8 times a week), fatty red meats and processed meats (less than 1 time a week), olive oil (more than 3 teaspoons a day), canola oil (regular consumption) and avocado (more than 3 units per week), wine (1 to 2 glasses per day, 4 or more days per week), sugar (less than 4 teaspoons per day), and snacks and/or sweetened beverages (no consumption). The total score ranged from 0 to 14 points. A score of <5 points indicates poor diet quality or low adherence to the Mediterranean diet; 5.5–8.5 points indicates average diet quality or moderate adherence to the Mediterranean diet; and >9 points indicates good diet quality or high adherence to the Mediterranean diet. The detailed algorithm for estimating the MDI score was presented in the original article.^16^ There was an adequate concordance between findings obtained by self-application of MDI and those achieved by the nutritionist, showing a correlation coefficient of 0.94 (p<0.001), a clinically insignificant mean difference of 0.14±0.56 total score between the two measurements, and Cohen’s kappa>0.61 for all items.^16^

### Risk and protective factors for non-communicable diseases

Information regarding smoking and physical activity was obtained using a validated questionnaire from the Chile’s National Health Survey (NHS) 2016-2017 (more information available in http://epi.minsal.cl/encuesta-ens-descargable/).

### Self-reported gingival health status and oral health habits

A validated SGH questionnaire^11 , 20^ was registered, including a 5-item instrument that assesses the frequency and performance of toothbrushing, the self-rate of gum health, presence of bleeding on toothbrushing, and of clinical signs of gingival inflammation. This instrument is reliable, presenting a Cronbach’s alpha of 0.73 and a Kappa-index ranging from 0.41 to 0.77 between the different questions.^20^ Dental attendance was registered using a validated questionnaire from the NHS 2016-2017 (more information available in http://epi.minsal.cl/encuesta-ens-descargable/).

### Statistical analysis

Based on non-probabilistic convenience sampling and considering a population universe of 18.95 million residents in Chile (2021), at a 95% confidence level and a 10% margin of error, the initial N was estimated as 97 individuals surveyed. Furthermore, a non-response rate of 10% was considered, resulting in a final N of 110 participants. The formula used to estimate the sample size was: $N_{e}=\left(z^{2}p(1-p)/e^{2}\right)/\left(1+\left(\left(z^{2}p(1-p)/e^{2}N\right)\right)\right)$ , where N_e_ was the estimated sample size, N was the sample universe, z was the Z score according to the desired confidence level, and e was the margin of error expressed as a percentage of decimals. The minimum sample size proposed for the SGH validation study was 138 was considered.^11^

The information obtained from PsyToolkit^17 , 18^ was downloaded into an Excel spreadsheet, where the anonymous database was processed. Statistical analysis was performed using STATA (17.0, StataCorp LLC, College Station, Texas, USA). Individuals with incomplete data were excluded from this study.

First, a descriptive analysis of the surveyed population was performed on sociodemographic variables, habits, MDI, and SGH. Data were described according to their distribution, obtained from the Shapiro-Wilk normality test and histogram observation. For normally distributed continuous variables, the mean and standard deviation were used, and for nonparametric variables, the median and interquartile range (IQR) were used. Absolute and relative frequencies were reported for categorical variables.

Fisher’s exact test was used to determine differences in the proportions of the study variables between the groups. The significant differences obtained in Fisher’s test for the variables of interest with sociodemographic background and/or smoking were explored using comparators for two independent samples, with the parametric statistic Student’s t-test, or Mann-Whitney U. For two or more independent samples, ANOVA statistic with Bonferroni correction, or alternatively, the Kruskal-Wallis test was used.

First, bivariate analysis was performed for the variables of interest, MDI, and SGH, using Spearman’s correlation analysis, with age, sex, educational level,^21^ visits to the dentist in the previous year,^22 - 24^ and smoking^25 , 26^ as predictors. Thus, the construction and validation of the directed acyclic graph (DAG) proposed for multivariate association analysis were performed using the aforementioned findings. Subsequently, a backward stepwise selection multivariate logistic regression model was performed for each SGH, eliminating variables with p≥0.2 and adding those with p<0.1 by an iterative likelihood ratio test estimation,^27^ reporting a crude, full (including all covariates), and adjusted model (including only the most significant covariates). The full model consisted of MDI (continuous) as a predictor of SGH (dichotomous), and age, sex, educational level, smoking, and dental attendance as confounders. The fitted models were subjected to diagnostics by a goodness-of-fit test (Lemeshow and Hosmer, 1982) and a post-estimation test (linktest) (Pregibon, 1980) to evaluate the specificity of the fitted regression model for each SGH. The output of the regression models was presented as odds ratios (OR) with 95% confidence interval (95% CI). Statistical significance was p<0.05.

## Results

Of the 404 people who entered the survey link in PsyToolkit and accessed the questionnaire, only 351 completed the questionnaire (sampling error of ±3.3%, over a maximum of ±4.8%, 86.9% of completion rate). The 53 incomplete data (13.1% non-response rate) were excluded from the final database since they lacked data for SGH. The time taken by the population to complete the survey was five minutes (interquartile range (IQR 3)).

### Characteristics of the population

Of the 351 individuals aged 30 years (IQR 21), 75.8% were female. Most of them had a university education (72.9%) and self-reported not having systemic diseases (78.9%). The other 21.2% self-reported having asthma (n=9), bipolar disorder (n=2), cancer (n=5), diabetes mellitus type 2 (n=15), epilepsy (n=2), hypertension (n=22), hypothyroidism (n=13), or autoimmune disorders or syndromes (n=14) (8 individuals reported combined diseases).

Regarding their habits, 55.56% of the participants self-reported never having smoked. Participants tended to brush their teeth two (50.43%), three or more (38.18%) times a day, doing it well (59.54%) or average (22.22%). A large proportion practiced physical activity at least once a week (n=170). Most participants reported having visited a dentist less than six months previously (40.17%) ( Table 1 ).

**Table 1:** Distribution of the study population according to sociodemographic variables, habits, and dental attendance

Variable		n	%
N° of adults aged 18-60 years old from the tripartite community of University of Chile		351	100
Age (Median, IQR)	30 (21) years		
Sex	Female	266	75.8
	Male	85	24.2
Educational level	Primary school	1	0.3
	Secondary school	50	14.3
	Technical	44	12.5
	University	256	72.9
Systemic diseases	Yes	74	21.1
	No	277	78.9
Smoking	Yes, one or more cigarettes per day	38	10.83
	Yes, occasionally (less than one cigarette per day)	26	10.26
	No, I stopped smoking	82	23.36
	No, I have never smoked	195	55.56
How many times a day do you brush your teeth?	Less than once per day	2	0.57
	Once a day	38	10.83
	Twice a day	177	50.43
	Three or more	134	38.18
How would you rate your toothbrushing?	Very good	60	17.09
	Good	209	59.54
	Average	78	22,22
	Bad	4	1.14
When was the last time you visited the dentist?	Less than six months ago	141	40.17
	Between 6 months and a year ago	59	16.82
	More than one year and less than two years ago	87	24.79
	Two or more, but less than five years ago	51	14.53
	Five or more years ago	11	3.13
	Never visited	2	0.57
Physical activity	Yes, three or more times per week	87	24.79
	Yes, once or twice per week	83	23.65
	Yes, less than four times a month	49	13.96
	I did not practice sports during the month	132	37.61

Mean MDI score was 8±2 points in total. For the poor diet quality category, the total score was 4 (IQR 0) for five individuals (1.42%). In total, 180 individuals (51.28%), whose diet quality was categorized as fair, obtained an MDI score of 7 (IQR 2). The remaining 166 individuals (47.29%) self-reported good diet quality, with a total MDI score of 10 (IQR 2).

Regarding SGH, for the question “ *How would you rate your gum health?”* 17.09% self-reported very good, 53.38% good, 25.64% average, and 3.99% bad. For *“During toothbrushing, do your gums bleed?”* , 2.56% self-reported always, 5.41% often, 43.59% sometimes, and 48.43% never. Finally, for the indicator *“In the last month. have you noticed your gums reddish and/or swollen?”* , 0.57% self-reported always, 4.27% often, 28.49% sometimes, and 66.67% never.

### Bivariate analysis

#### Adherence to the Mediterranean diet

Independent samples for the average MDI and sex were compared, finding a significant difference between Female (8.59±1.98) and Male (7.99±1.88), using Student’s t-test (p<0.05). Then, performing a Kruskal-Wallis test for educational level, significant differences were found in the MDI scores between high school (8 (IQR 2)) and university (9 (IQR 2)) (p<0.05).

Regarding smoking, no statistically significant difference was shown in the MDI score between the group that currently smoked (8.05±1.89) and the group that did not (8.55±1.98), according to Student’s t-test (p>0.05). Moreover, when the mean MDI of the group that visited the dentist in the previous year (8.46±2.07) was compared with the group that did not (8.42±1.83), there was no significant difference according to Student’s t-test (p>0.05).

## Self-reported gingival health status

Regarding sociodemographic variables and SGH, no statistically significant differences were found between age, sex, and binary categories of SGH (p>0.05). When the SGH categories were compared based on current smoking habit, significant differences were found between groups for all SGH (p<0.05) after applying Fisher’s exact test. However, when these differences were explored in the binary categories of each SGH using the Mann-Whitney U test, no significant differences were found between groups (p>0.05).

Moreover, there were no significant differences between the groups for dental attendance regarding all SGH (p>0.05).

### Mediterranean diet and self-reported gingival health status: relationship between outcome measures

On the other hand, statistically significant differences were found according to dietary quality and the questions *“During toothbrushing, do your gums bleed?”* (p=0.002) and *“In the last month, have you noticed that your gums are reddish and/or swollen?”* (p=0.004) using Fisher’s exact test. Furthermore, statistically significant differences were found between the total MDI scores for all the dietary quality categories (p<0.001). Then, using Student’s t-test, the MDI averages for each binary category of the SGH were compared. Differences in the MDI averages were found between groups for the indicator *“How would you rate your gum health?”* (dichotomous) (p=0.0016), *“During toothbrushing, do your gums bleed?”* (dichotomous) (p<0.05) and *“In the last month, have you noticed that your gums are reddish and/or swollen?”* (dichotomous) (p<0.001).

The contingency table for the bivariate analysis included the variables 1) *“How would you rate your gum health?”* (dichotomous), 2) *“During toothbrushing, do your gums bleed?”* (dichotomous), 3) *“In the last month, have you noticed that your gums are reddish and/or swollen?”* (dichotomous), 4) MDI (continuous), 5) age, 6) sex, 7) educational level, 8) dental attendance (dichotomous), and 9) smoking (dichotomous). The contingency table presents the rho coefficients and the significance levels of these relationships ( Table 2 ).

**Table 2:** Contingency table for the bivariate analysis using Spearman correlation

	1	2	3	4	5	6	7	8	9
1									
2	0.34**								
3	0.42**	0.36**							
4	0.15*	0.09	0.20**						
5	0.09	0.08	0.01	0.1					
6	-0.03	-0.03	0.03	-0.13*	0.03				
7	0.14*	0.09	0.2	0.15*	-0.07	0.01			
8	0.17*	0.08	0.12*	-0.01	0.02	-0.006	0.01		
9	0.08	-0.002	0.08	0.11*	-0.07	0.02	0.03	0.01	

*p<0.05, **p<0.001, p>0.05. Bold indicates statistically significant results

Note: 1) “How would you rate your gum health?” (dichotomous), 2) “During toothbrushing, do your gums bleed?” (dichotomous), 3) “In the last month, have you noticed that your gums are reddish and/or swollen?” (dichotomous), 4) MDI (continuous), 5) age, 6) sex, 7) educational level, 8) dental attendance (dichotomous), and 9) smoking (dichotomous).

## Multivariate regression analysis

**Figure 3:**
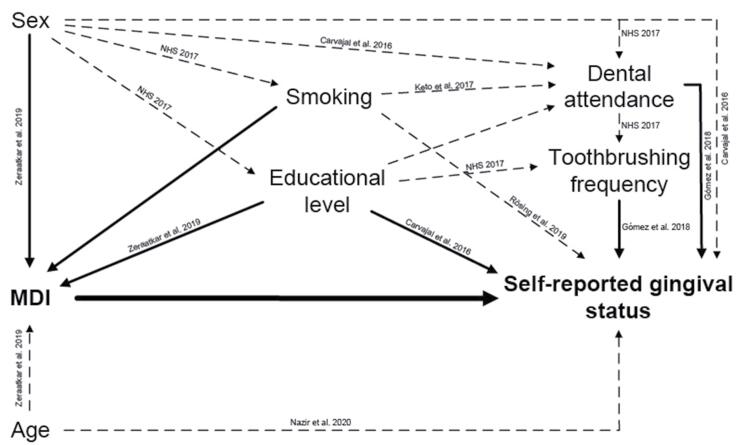
Directed acyclic graph proposed to evaluate the association between MDI score and SGH

To control possible confounders, the full multivariate model was adjusted for theoretical covariates (dashed line) and observed in bivariate linear correlation models (solid line), as shown in the DAG ( Figure 3 ).

Table 3 shows that the association between the MDI score and self-perception of gingival health status was significant, controlling for age, sex (p>0.05), educational level, and dental attendance (p<0.05), with an OR for the adjusted model of 1.18 (95% CI 1.04-1.34). However, the association between the MDI score and self-reported absence of bleeding due to brushing was significant with an OR of 1.12 (95% CI 1.01-1.25), adjusted for dental attendance. For the association between the MDI score and self-perception of signs of gingival inflammation, a significant association was found, which was not attenuated after adjusting for age, sex, educational level, and dental attendance in the last year, with an OR of 1.24 (95% CI 1.10-1.40) (p<0.001).

**Table 3:** Logistic regression models to evaluate the association between MDI score and SGH

Independent variables	How would you rate your gum health? (dichotomous) (OR [95% CI])			During toothbrushing, do your gums bleed? (dichotomous) (OR [95% CI])			In the last month, have you noticed your gums reddish and/or swollen? (dichotomous) (OR [95% CI])		
	Crude	Full	Adjusted	Crude	Full	Adjusted	Crude	Full	Adjusted
MDI score	**1.21 [1.07-1.37], p=0.002**	**1.18 [1.03-1.34], p=0.015**	**1.18 [1.04-1.34], p=0.013**	**1.12 [1.01-1.25], p=0.036**	1.10 [0.98-1.23], p=0.097	**1.12 [1.00-1.25], p=0.035**	**1.24 [1.11-1.41], p<0.001**	**1.23 [1.09-1.40], p=0.001**	**1.24 [1.10-1.40], p<0.001**
Age	1.01 [0.99-1.03], p=0.193	1.02 [1.00-1.04], p=0.127	1.02 [1.00-1.04], p=0.132	1.00 [0.99-1.03], p=0.275	1.01 [0.99-1.03], p=0.233	-	1.00 [0.98-1.02], p=0.908	1.00 [0.98-1.02], p=0.988	-
Sex (ref. Female)	0.86 [0.52-1.48], p=0.621	0.89 [0.51-1.55], p=0.691	-	0.87 [0.54-1.43], p=0.589	0.89 [0.54-1.48], p=0.676	-	1.10 [0.65-1.85], p=0.725	1.22 [0.71-2.11], p=0.463	-
**Educational level (ref. Primary school)**									
High- school	0.57 [0.3-1.57], p=0.08	0.62 [0.32-1.20], p=0.155	0.62 [0.32-1.20], p=0.158	0.69 [0.37-1.28], p=0.237	0.75 [0.40-1.39], p=0.360	-	0.58 [0.31-1.09], p=0.089	0.66 [0.35-1.26], p=0.212	-
Technic	**0.50 [0.26-0.97], p=0.04**	**0.48 [0.23-1.00], p=0.047**	**0.48 [0.23-1.00], p=0.049**	0.66 [0.35-1.26], p=0.211	0.61 [0.31-1.22], p=0.166	-	**0.51 [0.26-0.97], p=0.041**	0.57 [0.29-1.15], p=0.118	0.58 [0.31-1.13], p=0.11
Dental attendance (ref. more than a year)	1.32 [0.82-2.12], p=0.25	**2.06 [1.27-3.32], p=0.003**	**2.06 [1.27-3.32], p=0.003**	1.39 [0.91-2.13], p=0.124	1.36 [0.89-2.10], p=0.156	1.38 [0.90-2.11], p=0.143	1.28 [0.82-1.99], p=0.286	1.28 [0.81-2.03], p=0.288	-
Smoking (ref. yes)	**2.09 [1.31-3.32], p=0.002**	1.51 [0.86-2.66], p=0.152	-	0.99 [0.59-1.65], p=0.967	0.93 [0.55-1.58], p=0.152	-	1.49 [0.88-2.53], p=0.140	1.30 [0.75-2.25], p=0.350	-

Note: The university category was omitted because of estimability. Bold indicates a statistically significant association (p<0.05).

## Model fit

The p-values for the Hosmer-Lemeshow test in the adjusted models were greater than 0.1, indicating little evidence of poor goodness-of-fit. Regarding the post-estimation tests for the adjusted models, the test is based on the idea that if a regression-type equation is well specified (_hat<0.05), no additional independent variable should be significantly above the chance level (_hatsq>0.05), which was met for all the adjusted models ( Table 3 ).

## Discussion

### Key results and interpretation

Our findings support the proposed hypothesis and can be interpreted as follows: for each point where the MDI increases, there are 18% greater odds of self-reporting very good/good gingival health, 12% greater odds of self-reporting the absence of bleeding on toothbrushing, and 24% greater odds of self-reporting the absence of clinical gingival inflammatory signs, after adjusting for age, sex, educational level, dental visits in the previous year, and smoking.

Although this study only included an entirely web-based self-reported measurement of dietary and gingival health status, the findings are consistent with those of other studies conducted using heterogeneous methodologies. Randomized clinical trials (RCTs) conducted in Germany and India showed a reduction in clinical indicators after a Mediterranean-based intervention from 4 to 6 weeks.^4^ These findings are also in line with studies that have associated high adherence to the Mediterranean diet and a reduction in the odds of being diagnosed with Stage III/IV periodontitis in a university population.^28^ The results also contribute to observational studies that have evaluated other healthy dietary patterns in Germany, Brazil, Korea, United Kingdom, United States of America, Finland, and Jordan, in which adjusting for age, sex, and educational level showed that adults with better diet quality are less likely to have gingival bleeding and periodontitis.^4 , 29 - 32^ On the other hand, these findings are in line with a recently published cohort study from Italy, demonstrating that individuals with unhealthy lifestyles, i.e., low adherence to Mediterranean diet, low physical activity levels, high levels of stress, and poor sleep quality showed worse clinical outcomes three months after Steps 1/2 of periodontal therapy, that is, a worse response to the treatment.^33^

The path by which this association occurs may involve local and/or systemic mechanisms, or even epigenetic factors. First, the Mediterranean diet reduces the amount of periodontopathogen species in supragingival plaque.^34 , 35^ Moreover, molecules abundant in this diet, such as quercetin, present in several fruits and vegetables, or polyunsaturated fatty acids (PUFAs), such as omega-3, present in olive and canola oils, nuts, soybeans, tofu, and fish, could regulate inflammatory mechanisms relevant to the pathophysiology of periodontal diseases.^36 - 38^ These mechanisms are preceded by epigenetic modifications due to the Mediterranean diet, improving insulin metabolism and, consequently, preventing insulin resistance in non-diabetic patients^39^ and thus controlling the indirect etiopathogenesis of PDs.

### Strengths and Limitations

This is the first study in Chile to answer this research question using correctly specified and adjusted multivariate models based on statistical data from a representative sample of Chilean adults, particularly from a university community, collected using a low-cost and time-saving methodology. Thus, this study is a good approximation and precursor for future lines of research in Chile on the common dietary risk factors of NCDs, particularly PDs. However, these findings should be cautiously interpreted.

Online self-report surveys are low-cost and, in this study, a feasible methodology for conducting studies in complex circumstances. Consequently, they could be applied in a multicenter setting for active surveillance of PDs in the most affected populations in the southern cone, with a high burden of the disease; however, sometimes without resources to characterize, prevent, or treat it. In this sense, PsyToolkit^17 , 18^ allows an intuitive, complete, and dynamic coding and user interfaces, enabling the self-application and subsequent automatic estimation of complex algorithms such as the MDI, an indicator that can be a sufficient and useful diet screener in dental studies.^15^ Moreover, PsyToolkit has no response limits.^17 , 18^ Thus, probabilistic sampling can be performed for large populations without any major drawbacks. Therefore, one way to reach a larger and heterogeneous population and, consequently, improve the representativeness would be to incorporate this or another diet quality index, together with SGH, in future national health surveys or large-scale governmental surveys.

Regarding data analysis and interpretation, the models were adjusted for the main South American risk indicators reported, which are also the most used covariates in NCD-related nutritional epidemiology.^21^ The covariates used in our study were adequate for the proposed objective and expected internal validity. However, longitudinal studies are necessary to test the causal relationships proposed in the DAG, specifically using data that allows adjustment for other habits, such as oral hygiene habits or physical activity, to identify the impact of diet on periodontal health status as a risk factor.

It is essential to highlight some of the characteristics observed and explained by the convenience sampling strategy and recognize the fact that the collected data were from population most likely to complete online surveys.^40^ A large part of the population self-reported healthy lifestyles and habits, including regular physical activity, a high-quality diet, non-smoking habits, or effective toothbrushing performance. Furthermore, this population has regular access to dental care. These factors are associated with the educational level of our population,^41^ which involves an economic determinant of access to universities that biases the selection of participants.

On the other hand, we assumed that the university population has a high self-perception of PDs, possibly related to a greater concern and possibility of sustaining a healthy lifestyle, which corroborates the findings of the SGH. Thus, an essential aspect to evaluate is the self-perception of PDs in this and other populations. Studies that also consider this variable, such as the Brief-Illness Perception Questionnaire or the Illness Perception Questionnaire Revised for Oral Health,^42^ are necessary to identify SGH, which has a direct relationship with respondents’ identification of the clinical signs of gingival inflammation. Recently, self-reporting of bleeding caused by brushing, specifically the observation of a reddish coloration in white toothpaste foam, is a good diagnostic indicator of gingivitis and is easily detectable at the household level.^9^ However, this could be biased since smokers have less gingival bleeding given the local vasoconstriction generated by cigarette smoke.^25^ Unfortunately, this study lacked clinical examinations that confirm the periodontal/gingival health status, or the other self-reported conditions of the population, resulting in the existence of a high risk of detection bias. A more exhaustive validation of the SGH in adults at the national level is required, especially since the current form of the questionnaire used in this study is validated for the adolescent population only, constituting a limitation.

Furthermore, we used the dichotomous variable “visit to the dentist in the last year” as a surrogate variable for increased plaque removal,^22^ assuming that this would be reinforced by oral hygiene instruction or performed professionally at the same visit. However, we had no data on the nature of visits, whether for periodontal therapy, by public or private services, or if the person’s ability to stabilize their disease was evaluated, or whether dietary counseling for PDs prevention was provided during these visits. However, we assumed that almost half of the professionals would perform some type of nutritional care, for example, with dietary analysis and subsequent nutritional or dietary counseling.^23^ Studies are needed that also specify this information at the national level as well as the circumstances and reasons for performing such counseling.

### Future research directions

Further research, either RCTs or complementary real-world observational studies, are required to determine the effect of healthy diets or the overall dietary pattern on PDs prevention in patients with or without other associated NCDs. Although diet could also impact signs of periodontal disease that affect dental support,^4^ this study was designed particularly for gingival inflammation. Future investigations could include signs of periodontal destruction, accompanying the diet survey with a complete clinical periodontal examination, considering that in older people and in smokers the absence of inflammation may not be the absence of periodontal disease. Furthermore, healthy diet interventions should be compared with standard periodontal treatment^43^ to inform and develop algorithms for low-cost and timesaving surveillance and risk characterization of periodontal diseases in Chile and Latin America, using the “big data.” Similarly, although initially elucidated, it is necessary to determine the possible pathways related to local, systemic, or epigenetic modulation by which a Mediterranean or healthy diet affects periodontal tissues, which justifies conducting this research in populations with different genetic heritage.

## Conclusion

We associated adherence to the Mediterranean diet with better self-reported gingival health status in a population of Chilean adults in an entirely web-based research environment. Longitudinal studies with random sampling are required to establish the effect of diet on gingival and periodontal health. Nevertheless, this evidence could contribute to the design of low-cost surveillance programs to reduce the burden of periodontal disease and related “common risk factors”.
